# SEA-mulation: training for technical treatment related to emergency situations in maritime environment

**DOI:** 10.1186/s13054-019-2599-y

**Published:** 2019-09-11

**Authors:** Brice Loddé, Gael-Emgan Querellou, Alexandre Morel, Erwan L’Her

**Affiliations:** 1 Université de Bretagne Occidentale, CS 93837, 29238 Brest Cedex 3, France; 2 Service de Santé au Travail et Maladies liées à l’environnement, CHRU Morvan, 29609 Brest Cedex, France; 3 Société Française de Médecine, UFR Médecine et Sciences de la santé, 22, Avenue Camille Desmoulins, 29200 Brest, France; 4 Centre de Simulation en Santé, UFR Médecine et Sciences de la Santé, 22, Avenue Camille Desmoulins, 29200 Brest, France

As Schell and coll [1] wrote in their article dealing with “The global need for essential emergency and critical care”, *critical illness can occur in anyone irrespective of age, gender, or social status, it can begin in the community or in hospital, and does not respect traditional divisions into medical specialties*.

We can add that those conditions could be applied to critical life-threatening accidents or critical acute poisonings as well.

If the environment where can occur such health troubles isolates the victims, it takes part in the difficulty of caring the affected patients. And that is particularly the case for the maritime environment.

In fact, emergency medical assistance at sea can be quite complex for four main reasons.

Firstly, there could be many victims, be they crew members or passengers, who quickly find themselves in critical emergency situations, as when the *Costa Concordia* capsized.

Secondly, health care capacity on board, principally represented by a medicine chest and seafarer-training in first aid (with additional telemedical advice), is often insufficient in an emergency situation which requires a high degree of technical accuracy [2].

Thirdly, the position of ships far from onshore health facilities often requires bringing a medical team as quickly as possible to prepare, stabilise and transfer the patient(s) to hospital [3].

Fourthly, ships (except those with a sick bay) can be difficult places to treat people, as they are subject to wave motion which hampers care interventions.

In response to these critical points, the medicine simulation centre of the Brest Medicine Faculty aims at training healthcare professionals involved in such situations.

The principle of this training could be referred in what Brotherton and coll [4] expressed in their article upon “ECCCOing the call for emergency and critical care training in low middle-income countries”: *clinical care, teaching and research are key in understanding what works and what is sustainable*.

Thereby, the objectives of training are not only gaining skills into high technicality therapeutic interventions in isolated backgrounds but also facing the complexity of performing them in an environment copying rough sea conditions [5].

Indeed, besides the usual high-fidelity dummy patients, the Brest Medicine simulation centre has been equipped with a vibratile platform with variable (frequency and acceleration intensity), surrounded with screens displaying 220° videos of boat or helicopter insides, of unsettled skies and seas, thus creating a training environment to get accustomed to life at sea.

Moreover, adding on-board noises and smells enables the platform to reproduce professional situations more genuinely. The expertise being defined according to specific situations, getting trained in typical conditions close to those met during sea operations, results in developing skills necessary to healthcare professionals who intend to work in embarked medicine or at sea emergencies.

Besides, in other structures, a different sort of simulator is used for the training to winch accidents: the HUET (Helicopter Underwater Escape Training) during which the trainees are tied to their seats inside a helicopter cockpit immerged in the water, learn to free themselves out and by so doing get to know how to react accurately when an accident involving a rescue craft occurs.

By acquiring a more comprehensive training through these two types of simulators, winched-up healthcare professionals will be able for a certainty to achieve the right procedure in case of emergency.

## Data Availability

All the material and the data described in the present manuscript belong to the simulation centre of Brest Medicine Faculty.
